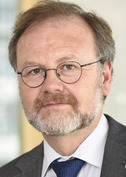# Boron: Its Role in Energy‐Related Processes and Applications

**DOI:** 10.1002/anie.201911108

**Published:** 2020-04-06

**Authors:** Zhenguo Huang, Suning Wang, Rian D. Dewhurst, Nikolai V. Ignat'ev, Maik Finze, Holger Braunschweig

**Affiliations:** ^1^ School of Civil & Environmental Engineering University of Technology Sydney 81 Broadway Ultimo NSW 2007 Australia; ^2^ Department of Chemistry Queen's University Kingston Ontario K7L 3N6 Canada; ^3^ Institute for Inorganic Chemistry Julius-Maximilians-Universität Würzburg Am Hubland 97074 Würzburg Germany; ^4^ Institute for Sustainable Chemistry & Catalysis with Boron (ICB) Julius-Maximilians-Universität Würzburg Am Hubland 97074 Würzburg Germany; ^5^ Merck KGaA 64293 Darmstadt Germany

**Keywords:** boron, electrolytes, hydrogen, OLEDs, small-molecule activation

## Abstract

Boron's unique position in the Periodic Table, that is, at the apex of the line separating metals and nonmetals, makes it highly versatile in chemical reactions and applications. Contemporary demand for renewable and clean energy as well as energy‐efficient products has seen boron playing key roles in energy‐related research, such as 1) activating and synthesizing energy‐rich small molecules, 2) storing chemical and electrical energy, and 3) converting electrical energy into light. These applications are fundamentally associated with boron's unique characteristics, such as its electron‐deficiency and the availability of an unoccupied p orbital, which allow the formation of a myriad of compounds with a wide range of chemical and physical properties. For example, boron's ability to achieve a full octet of electrons with four covalent bonds and a negative charge has led to the synthesis of a wide variety of borate anions of high chemical and electrochemical stability—in particular, weakly coordinating anions. This Review summarizes recent advances in the study of boron compounds for energy‐related processes and applications.

## Graphical abstract

**The fifth element**: Boron has a unique position in the Periodic Table—directly at the apex of the line separating metals and nonmetals. This Review highlights the critical role of boron and boron compounds in the fields of energy conversion and storage, and demonstrates the versatility and potential of boron for energy‐related research.

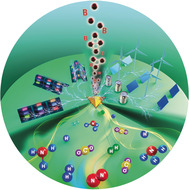

## 1 Introduction

In terms of energy‐related research, the element boron is currently enjoying significant attention from scientists working in various fields. Most notably, recent advances in the fundamental understanding of boron chemistry and breakthroughs in the synthesis of its compounds have seen boron playing an increasingly important role in applications such as small‐molecule activation for fuel synthesis, organic light‐emitting diodes (OLEDs), hydrogen production and storage, and electrolyte materials. The rich applications of boron‐containing compounds are closely associated with the unique properties of the element itself. For example, from an electronic structure point of view, boron's electron deficiency means that many of its compounds can act as electrophiles and/or Lewis acids;^1^ while under certain conditions the atom can be negatively charged or polarized and, therefore, behave like a nucleophile or Lewis base.^2^ This flexibility allows boron to form a great variety of compounds with tunable properties for specific applications. For example, boron and hydrogen form a large number of boranes and anions, and their high hydrogen capacity make them potential candidates for hydrogen storage materials.^3^ A wide range of bulky and unsymmetrical borate anions and anionic boron clusters have also attracted interest because of their inherently weak affinity to cations, which makes them highly important building blocks for electrochemical devices.^4^

This Review highlights several aspects of boron‐containing compounds for energy‐related research, including small‐molecule activation, hydrogen storage, electrolytes, and OLEDs, with the aim of emphasizing the diverse roles and high potential of this element. Each topic starts with a short introduction followed by details of selected examples and discussion, and then closes with a brief perspective. Compared with carbon‐based materials, which have been explored extensively for energy‐related research, boron has attracted much less attention. This is likely because, in comparison with boron, it is much easier to control the morphology and structure and consequently the properties of carbon materials. This Review aims to highlight boron's ability to facilitate the development of efficient and economical methods and materials for future energy needs, and illustrates the great potential of this element to play an important, if not decisive, future role in this challenge.

## 2 Small‐Molecule Activation with Molecular Boron: Scratching the Surface

The reaction of transition‐metal complexes with dihydrogen is an extremely well‐established process, which forms the basis of both the practice and pedagogy of catalysis. In contrast, for centuries, main‐group compounds were thought to be unable to undergo reaction with H_2_ under mild conditions. This paradigm was abruptly shattered in the mid‐2000s, with the first uncatalyzed reactions of H_2_ with main‐group molecules under ambient conditions, namely with a digermyne in 2005,^5^ an ambiphilic phosphine/borane compound in 2006,^6^ and a cyclic (alkyl)(amino)carbene in 2007.^7^

The combination of filled and empty orbitals that are close in both space and energy is considered the key property of transition metals that allows them to interact with, and activate, relatively inert small molecules. In 2010, Power crystallized this concept in a short review article in *Nature* entitled “Main‐group elements as transition metals”, wherein a parallel was drawn between the filled/occupied orbital arrangements of transition metals and low‐oxidation‐state or multiply bound main‐group species.^8^ The results of the interceding years, particularly in the field of low‐valent boron chemistry, have made this review appear particularly prescient. In a recent article^9^ in *Chemical Reviews* we attempted to comprehensively encapsulate the various ways that hypovalent boron species are able to mimic the reactivity of transition‐metal species, particularly with regard to the activation of small molecules.

**Figure 1:**
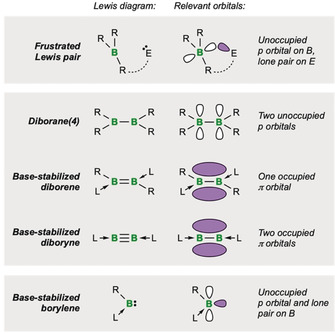
Schematic representation of the classes of molecular boron species described in this section.

Herein we provide a short overview of the abilities of low‐valent boron species to activate (or cooperate to activate) small molecules of interest to catalysis and the sustainable use of energy and resources. These processes are in general promoted by three distinct systems based on boron (Figure 1): 1) heterodinuclear activating species, namely frustrated Lewis pairs (FLPs), which function by the combination of a Lewis‐acidic boron component with a Lewis base; 2) homodinuclear activating species with two connected boron atoms, such as diboranes, diborenes, and diborynes; and 3) monoboron species, namely monovalent, dicoordinate borylenes.

### 2.1 Cooperative Heterodinuclear Activation: Boron Plus Lewis Base

The concept of frustrated Lewis pairs has truly captured the attention of chemists and beyond, and has led to an astounding number of reviews and books on the topic.^10^ The discovery by Stephan and co‐workers in 2006 of reversible dihydrogen splitting across a phosphine/borane system marked the initiation of the field of FLP chemistry.^6^ Since that time FLP‐mediated activations of a range of small molecules have been reported, including CO_2_, CO, N_2_O, NO, SO_2_, silanes, hydroboranes, olefins, dienes, cycloalkanes, and alkynes. Recently, reactions of strongly Lewis‐acidic boranes with N_2_‐containing molecules, namely transition‐metal dinitrogen complexes^11^ and diazoalkanes,^12^ have also been reported, spurring speculation into the possibility of FLP‐based dinitrogen activation.^13^ This may indeed be possible if the right combination of strong Lewis base and acid can be found; however, such reactivity remains, at present, speculative.

**Figure 2:**
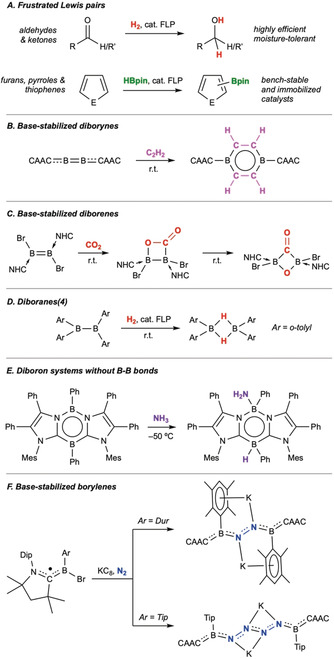
Selected recent highlights of boron‐based small‐molecule activation. NHC=N‐heterocyclic carbene, CAAC=cyclic (alkyl)(amino)carbene, Dur=2,3,5,6‐tetramethylphenyl, Tip=2,4,6‐triisopropylphenyl.

The maturity of the field and the obvious applicability of the systems for small‐molecule activation makes FLPs by far the most catalytically relevant boron species for this purpose. The hydrogenation of simple multiple‐bond‐containing (C=C, C=N, C=O) species was the first and most obvious catalytic use of FLPs, considering their facile dihydrogen splitting abilities. However, a range of other catalytic reactions have now been reported, such as S−H bond borylation, carbocyclizations, CO reduction with syngas, and CO_2_ hydrogenation. Recent advances have seen these catalytic procedures become even more attractive through increased efficiencies,^14^ water‐tolerance (**A**, Figure 2),^15^ and even the development of highly enantioselective conversions.^16^ Perhaps the most exciting recent development in FLP catalysis is the intermolecular C(sp^2^)−H bond activation and borylation of heteroarenes reported by Fontaine and co‐workers.^17^ By using an intramolecular FLP based on a 1‐amino‐2‐borylbenzene backbone as the catalyst, the authors were able to borylate a range of electron‐rich heteroarenes in excellent yields—a process that has since been extended to include bench‐stable and solid‐immobilized precatalysts (**A**, Figure 2). As catalysis based on intermolecular C−H activation is highly desirable but also extremely challenging to achieve, these advances are particularly promising.

### 2.2 Cooperative Homodinuclear Activation: Systems with B−B Bonds

At first glance, compounds with B−B single, double, or triple bonds^18^ do not seem particularly well‐suited to small‐molecule activation. Given that acceptance of electron density from a bond is often required to break bonds in small molecules, an empty orbital is usually required in the activating system. Although base‐stabilized diborenes and diborynes do not possess unoccupied p orbitals like the FLP‐based systems (see Section 2.1) and borylenes (see Section 2.3) described herein, a number of other vacant orbitals exist that can potentially accept electron density from an incoming small molecule. These include the antibonding π*(BB) orbital(s) and partially vacant p orbitals from attached π‐accepting ligands (e.g. cyclic (alkyl)(amino)carbenes; CAACs).

Carbon monoxide serves as an excellent test case for small‐molecule activations, and this molecule has shown diverse reactivity with diborenes and diborynes. These reactive species not only bind CO, but also lead to its reduction, coupling, and even scission.^19^ Diborynes have been shown to undergo hydrogenation,^20^ albeit reluctantly in many cases, while they readilly activate C−H bonds of acetone,^21^ and cyclize and activate C−C bonds of alkynes (**B**, Figure 2).^22^ Diborenes, in some respects more reactive alternatives to diborynes, have been shown to undergo inverse‐electron‐demand Diels–Alder reactions with dienes,^23^ and bind and cleave one C=O bond of CO_2_ (**C**, Figure 2).^24^

An interesting recent advance is the growth of boron‐based systems for small‐molecule activation with only B−B single bonds or even two boron atoms not directly bonded to one another. Traditional diboranes(4) present the opposite case from diborenes and diborynes: they possess the requisite empty p orbitals, but lack the filled π orbitals or lone pairs of the other species described herein. In this regard, some surprising activations of dihydrogen (and CO) have been reported with a range of singly bonded species, such as a tetraaryldiborane(4) (**D**, Figure 2), a diaryl, dithieno‐bridged diborane(4), and a borylborenium cation.^25^ A number of electron‐rich diboron systems without B−B bonds have not only shown reactivity with dihydrogen but also with other small molecules such as ethylene, alkynes, O_2_, and even NH_3_ (**E**, Figure 2).^26^

### 2.3 Activation at a Single Boron Atom: Borylenes

Borylenes of the form [:BR] are highly reactive species with both a lone pair of electrons and an empty p orbital on boron, and as such have never been isolated in their free form. Early efforts towards generating and trapping borylene species hinted at their impressive ability to activate small molecules. The respective studies of Timms as well as Pachaly and West led to the reliable generation of transient borylenes and their trapping with alkanes, alkynes, and cyclic ethers by C−H and C−O insertion reactions.^27^ Recent studies from the group of Bettinger have shown that transient borylene species can be generated photolytically and react under matrix conditions with a range of small molecules such as CO, methane, ethene, ethyne, and even N_2_.^28^

For a long time, attempts to harness borylene reactivity under condensed‐phase conditions were limited to transition‐metal borylene complexes,^29^ as stabilized and synthetically convenient equivalents of “free” borylenes. Transition‐metal borylene complexes show diverse binding and reactivity patterns with CO and other donors, such as isonitriles and azides, leading in some cases to metal‐free, doubly base‐stabilized borylene species.^30^

The discovery by Bertrand and co‐workers in 2010 of a synthetic route to doubly base‐stabilized borylenes opened a new chapter in borylene chemistry, which provided access to isolable borylenes stabilized not through boron–metal interactions but through Lewis‐base stabilization.^31^ Since that time, a range of metal‐free borylene species have been prepared. However, examples of true metal‐free small‐molecule activation by borylene species are still limited. The most well‐defined and “truest” isolable borylene species is likely a dicoordinate, singly base‐stabilized aminoborylene isolated by Stephan, Bertrand, and co‐workers.^32^ Although linear in the solid state, this borylene activates both H_2_ and CO at room temperature, thus illustrating some of the most clear‐cut borylene‐based examples of small‐molecule activation by isolated species.

Our work in this area led to the synthesis of two CO‐stabilized borylene species that showed distinct metal‐like photodecarbonylation reactivity.30b, 30d Depending on the reaction conditions, either intramolecular C−C activation or Lewis‐base addition was observed. This work led to the development of chemistry centered around a transient, reduction‐generated borylene species. Reduction of monohaloboron radical species of the form [(CAAC)BBrAr]^.^ (Ar=Mes or Dur) led to the putative generation of the dicoordinate borylenes [:BAr(CAAC)], which under an atmosphere of dinitrogen and further reduction led to either dipotassium di‐ or tetranitrogen complexes, depending on the nature of the aryl group at boron (**F**, Figure 2).^33^ Further treatment of these metalated compounds with water provided the respective diprotonated derivatives, thus providing the first binding and reduction of dinitrogen at a p‐block element.

### 2.4 Summary and Perspectives

Frustrated Lewis pairs, being the most developed of the species described herein, are clearly advanced in terms of applications in small‐molecule activation, with catalytic reactions now abundant and becoming increasingly efficient and practical. The stoichiometric reactions of low‐valent mono‐ and diboron species presented herein, while far from having any practical or large‐scale utility, do show an impressive ability to bind and activate small molecules. This hints at a very interesting future for metal‐free reactions of boron and, by extension, other main‐group elements that may have been historically overlooked for various reasons.

## 3 Boron Compounds for Hydrogen Storage

Boron is able to form a wide range of hydrogen‐rich molecules, such as boranes and borohydrides. As a consequence of their high hydrogen capacity by weight, these compounds have been considered as hydrogen carriers. Hydrogen has a specific energy that is much higher than common carbon‐based fuels, but also a very low energy density by volume.^34^ Hydrogen storage has become the bottleneck for the wider deployment of hydrogen fuel cells in cars, which require a method featuring both the high volumetric and gravimetric densities of hydrogen. Conventional methods of compression and liquefaction offer quite low density, and they are further penalized by the energetic cost of converting H_2_ gas into these physical states. Material‐based hydrogen storage has thus attracted attention, with boranes and borohydrides being intensively studied.^3^

### 3.1 Amine boranes

Interest in amine boranes for hydrogen storage is primarily driven by two factors: 1) their high hydrogen capacity and 2) their low hydrogen release temperature. The high capacity is associated with their molecular compositions, wherein the light nitrogen and boron atoms typically bond with multiple hydrogen atoms. Amine boranes bear protic (N‐H) and hydridic (B‐H) hydrogen atoms in proximity, thereby leading to dihydrogen interactions that are conducive to H_2_ formation.3a

**Figure 3:**
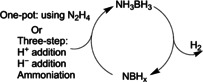
Hydrogen evolution by thermolysis of ammonia borane and its regeneration.

One of the best‐known compounds for hydrogen storage is ammonia borane (Figure 3, NH_3_BH_3_, commonly known as AB), which has a high capacity of 19.6 wt %.^35^ The release of the chemically bound H_2_, however, is often accompanied by the formation of impurities such as borazine and ammonia. The actual thermal decomposition of pure AB involves several steps, namely, induction, nucleation, and growth, which are directly related to the disruption of the dihydrogen bonds, the formation of the diammoniate of diborane, [NH_3_BH_2_NH_3_][BH_4_] (DADB), and the bimolecular reaction between DADB and AB, respectively.35c Together with volatile gaseous products, linear and cyclic molecules are formed, and these are transformed into polymeric iminoborane as the temperature increases. Similarly, DADB has also been observed as a key intermediate when AB decomposes in ionic liquids or organic solvents.35d Notably, the presence of ionic liquids seems to effectively reduce the induction and accelerate the reaction kinetics. The derivatives MNH_2_BH_3_ (M=Li, Na) have been synthesized and display improved kinetics, reduced decomposition temperature, less exothermic reactions, and negligible impurities. All these can be ascribed to the change in the electronic state of the nitrogen atom caused by the substitution of H by electron‐donating metals.35a

Dehydrogenation has been performed in organic solvents, but typically fewer than three equivalents of H_2_ can be derived because of the formation of amino‐ or iminoboranes, BNH_*x*_. Metal‐based catalysts and Lewis or Brønsted acids have been studied to improve the dehydrogenation rate and capacity.^36^ For example, a catalyst of a Ni‐NHC complex, obtained by reacting bis(cyclooctadiene)nickel with N‐heterocyclic carbenes (NHCs), facilitates the release of 2.5 equiv H_2_, and this is likely associated with the strong donating capacity of the NHC, which activates the B−H bonds.36c Note that, compared with catalysts using expensive metals, Fe‐based catalysts are particularly interesting, since they will be cost‐effective for large‐scale application.36d

The typical spent fuel of AB consists of a mixture of polymers featuring strong B−N bonds, including polyaminoborane (NH_2_BH_2_)_*n*_ and polyiminoborane (NHBH)_*n*_. For practical application of AB as a hydrogen carrier, it is critical, but challenging at this stage, to regenerate AB in a cost‐effective way. The typical regeneration (Figure 3) involves three steps: 1) the digestion of spent fuel to form BX_*n*_ (X=anionic counterion), 2) the formation of BH_3_ using a reducing agent, and 3) the addition of NH_3_.^37^ The whole process would require significant energy, and coupling with renewable energy sources could be an option. One‐pot regeneration has been demonstrated, whereby hydrazine was used as both a digesting agent and a reducing agent.^38^ The production of hydrazine is, therefore, a topic of discussion to make this process viable for large‐scale application.

Hydrolysis has also been performed to drive hydrogen evolution. A catalyst is typically needed to promote the reaction, since AB is relatively stable in water. Heterogeneous catalysts such as metal nanoparticles are commonly used to facilitate combination between hydridic H atoms in AB and protic H atoms in water.^39^ The resulting hydrolytic borate products, however, possess unfavorable thermodynamics for the regeneration of borane.

### 3.2 Light‐Metal Borohydrides

**Figure 4:**
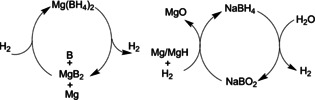
Partial regeneration of borohydride.

Borohydrides with light cations such as Li^+^, Na^+^, and Mg^2+^ have been considered for hydrogen storage.^40^ Their thermolysis, however, requires high temperatures, typically above 250 °C, for dehydrogenation. The resulting stable borides and elemental boron also make it highly challenging to reform borohydrides under mild conditions. Jensen and co‐workers achieved the direct hydrogenation of MgB_2_ to Mg(BH_4_)_2_ under 950 bar H_2_ at 400 °C.^41^ Recently, they found that after MgB_2_ has been ball‐milled with THF and Mg (or MgH_2_), it can be hydrogenated to Mg(BH_4_)_2_ at 300 °C under 700 bar of H_2_ (Figure 4).^42^ However, this process still requires a very high energy input, and other decomposition products of borohydrides such as amorphous boron have not been considered.

NaBH_4_ has been extensively studied as a hydrogen source for catalyzed hydrolysis, which leads to high‐purity H_2_ at room temperature. The low solubility of both NaBH_4_ and its hydrolytic products in water, however, necessitate the use of large amounts of water for effective hydrogen evolution, which eventually reduces the hydrogen capacity and, therefore, makes this system unsuitable for large‐scale applications. Regeneration of NaBH_4_ from the spent fuel involves breaking strong B−O bonds (dissociation energies: 193 kcal mol^−1^), which indicates it is a high‐energy process.^37^

In a regeneration method reported recently, magnesium was ball‐milled with NaBO_2_⋅2 H_2_O to produce NaBH_4_ (Figure 4). This process avoids the dehydration process to make NaBO_2_ and requires no expensive MgH_2_; therefore, it is comparably low‐cost.^43^

### 3.3 Octahydrotriborates

This family of compounds, sometimes known as triborates, containing the B_3_H_8_^−^ anion, are less well‐studied than borohydrides, likely because they are not readily available. Recent progress in synthesis allows the preparation of these compounds to be carried out in a typical chemistry laboratory.^44^ Generally speaking, as the number of B and H atoms increases, the less reactive the hydridic H atoms in the B_*m*_H_*n*_^*x*−^ anions become as a result of the increasing charge distribution across the large cluster. This is evident by NaB_3_H_8_ being much more stable and soluble in water than NaBH_4_,44b which means that less water is needed to prepare a liquid phase. Finally, its boron‐rich nature leads to the formation of various polyborates during hydrolysis that are relatively soluble in water, thus reducing the formation of precipitate.44c The NaB_3_H_8_ system, therefore, outperforms NaBH_4_ in terms of the hydrolytic generation of hydrogen. The regeneration of B_3_H_8_^−^ from the polyborates is yet to be demonstrated.

A few other B_3_H_8_^−^ compounds, such as [(NH_3_)_2_BH_2_][B_3_H_8_] and NH_4_B_3_H_8_, have also been investigated.44d, 44f Compared with borohydrides, B_3_H_8_^−^ compounds are less thermally stable and can decompose at temperatures below 100 °C. The collapse of the B_3_H_8_^−^ fragment sometimes generates volatile species such as B_2_H_6_ and B_5_H_9_, which are highly flammable and toxic. These volatiles can be suppressed, as seen in the case of C(NH_2_)_3_B_3_H_8_, which exists as a liquid under ambient conditions as a result of the large size of both the cation and anion.44e The superior mobility of the cation in a liquid state enables more efficient reaction between the protic (N‐H) and hydridic (B‐H) hydrogen atoms, effectively limiting the formation of B_2_H_6_ and B_5_H_9_.

### 3.4 BCN Heterocyclic Compounds

**Figure 5:**
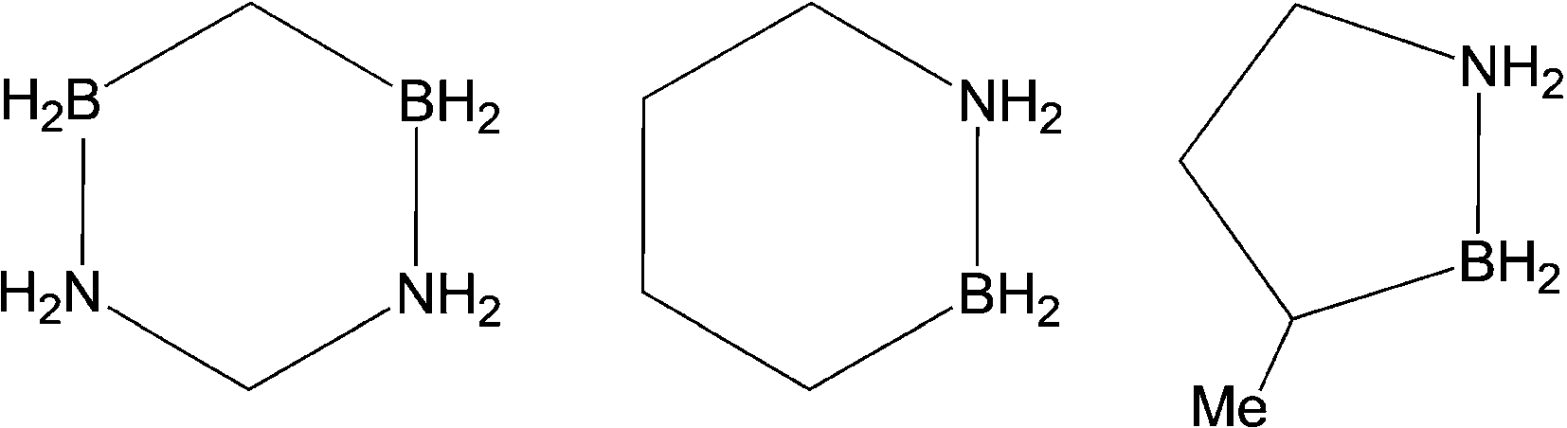
Some C‐, B‐, N‐containing heterocyclic compounds investigated for hydrogen storage.

From the hydrogen storage perspective, an interesting new class of compounds, namely carbon‐, boron‐, and nitrogen‐containing cyclic compounds, have been successfully synthesized and studied. These types of compounds were designed to take the form of some well‐known hydrocarbons such as cyclopentane and cyclohexane, but feature much improved hydrogen evolution. Under the mild operating conditions required for practical applications, the hydrogen capacity is determined by the reaction between the protic (N‐H) and hydridic (B‐H) hydrogen atoms. Liu and co‐workers have made significant contributions to the discovery of several CBN compounds for hydrogen storage (Figure 5).^45^ For example, bis‐BN cyclohexane, an isostere of cyclohexane with two BN units, has good thermal stability up to 150 °C, but it rapidly gives off pure H_2_ at room temperature in the presence of a catalyst.45a BN‐methylcyclopentane is a liquid that can release 2 equiv H_2_ and the exclusive trimer spent fuel can be converted back into the starting material in good yields.45b 1,2‐BN‐cyclohexane, also cleanly forms a trimer upon releasing H_2_. A single compound as the spent fuel that maintains the heterocyclic structure of the starting materials could be highly advantageous for regeneration, compared to the various polymeric compounds formed during the thermal decomposition of ammonia borane. The hydrogen capacity of these BCN systems is still low and their regeneration requires two steps, a digestion reaction to form ‐NH_2_ followed by a reduction reaction to form ‐BH_2_.45b

### 3.5 Summary and Perspectives

While no single compound is so far able to reversibly store and release the desired amount of hydrogen with minimum energy input and high efficiency, much has been learned through these studies. The attendant breakthroughs in the synthesis of new molecules and new synthetic methods have enriched the repertoire of methods for the exploration of hydrogen‐storage materials. The large variety of hydrogen‐rich boron‐containing units/compounds, either by themselves or coupled with other units, offer fertile ground for research.

## 4 Borate Anions and Anionic Boron Clusters as Building Blocks for Energy‐Related Materials

Liquid electrolytes are key components of many electrochemical devices used for the production of renewable energy for energy conversion, storage, as well as transportation. Li‐ion batteries are the current basis for mobile electrical energy and they are an important example of electrochemical devices based on liquid electrolytes.^46^ The development of Li‐ion batteries in the 1980s and their commercial availability since 1991 was crucial for the spread of portable devices such as mobile phones and notebooks. The capacity of commercial Li‐ion batteries is often not sufficient, and they are not ideal with respect to the requirements of a secure power supply, for example, for electrical vehicles. New materials with improved properties are thus needed for mobile energy storage devices. Various battery systems based on Li‐, Na‐, Mg‐ and other metal‐oxygen, ‐sulfur, and ‐air batteries are under development for mobile applications and flow batteries for stationary use.46a, 46b, 46c, 46e, 47 Supercapacitors (supercaps) are a further important class of electrochemical devices for energy storage, which, similar to batteries, rely on electrolytes as key components.^48^ A third example of electrochemical devices that often contain liquid electrolytes are dye‐sensitized solar cells (DSSCs, Grätzel cells).^49^

**Figure 6:**
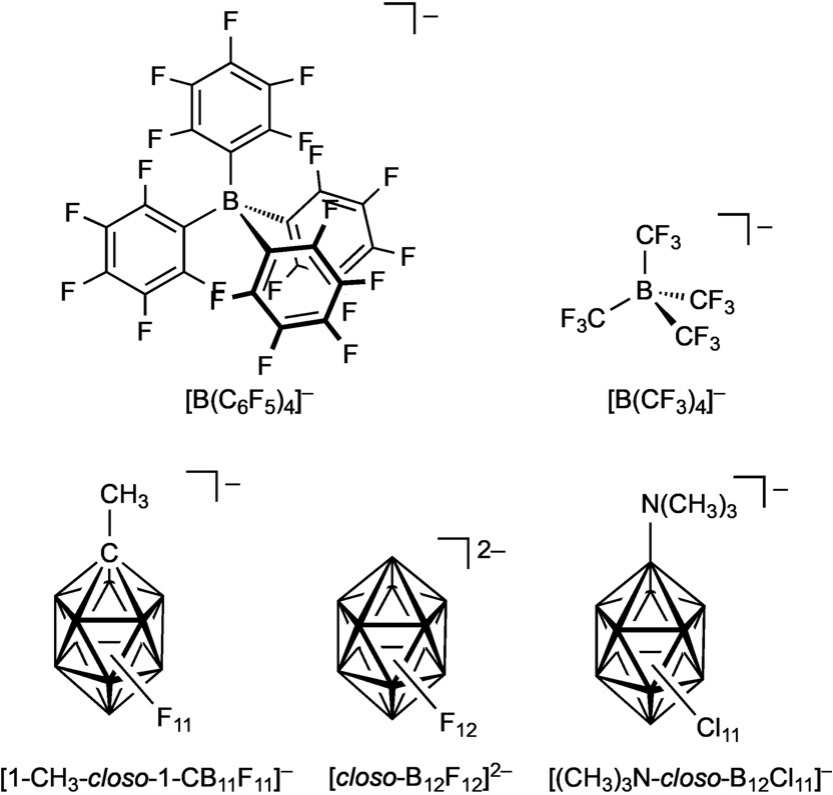
Examples of boron‐based WCAs.

Borate anions and negatively charged boron clusters are widely applied building blocks for liquid electrolytes used in the aforementioned electrochemical devices. Both types of boron‐based ions belong to the class of weak‐ to medium‐coordinating anions that have been used for the stabilization of highly reactive cations.^50^ In particular, anions with halogen substituents bonded to boron and anions with perfluorinated substituents are among the weakest coordinating anions (WCAs) that have been intensively studied and applied. Figure 6 shows examples of polyhalogenated WCAs that are either tetracoordinate borate anions (e.g. [B(C_6_F_5_)_4_]^−^,^51^ [B(CF_3_)_4_]^−[52]^) or are some of the most stable known mono‐ and dianionic 12‐vertex boron clusters (e.g. [1‐CH_3_‐*closo*‐1‐CB_11_F_11_]^−^,^53^ [*closo*‐B_12_F_12_]^2−^,^54^ and [(CH_3_)_3_N‐*closo*‐B_12_Cl_11_]^−[55]^).

A weak interaction between the cations and anions underpins a high ion mobility—one of the requirements for salts used as an electrolyte or as a component of an electrolyte composition used in electrochemical devices. A further prerequisite is a sufficient chemical, electrochemical, and thermal stability of these salts.

In this section we provide a brief survey of salts with borate anions and negatively charged boron clusters as building blocks of electrolytes for electrochemical devices. The first section contains an overview of salts with tetracoordinate borate anions that are relevant to battery applications. The second part focuses on cyanoborate anions as building blocks for low‐viscosity ionic liquids and their use in electrochemical devices. The third paragraph summarizes energy‐related applications of anionic boron clusters.

### 4.1 Borate Anions: Battery Applications

**Figure 7:**
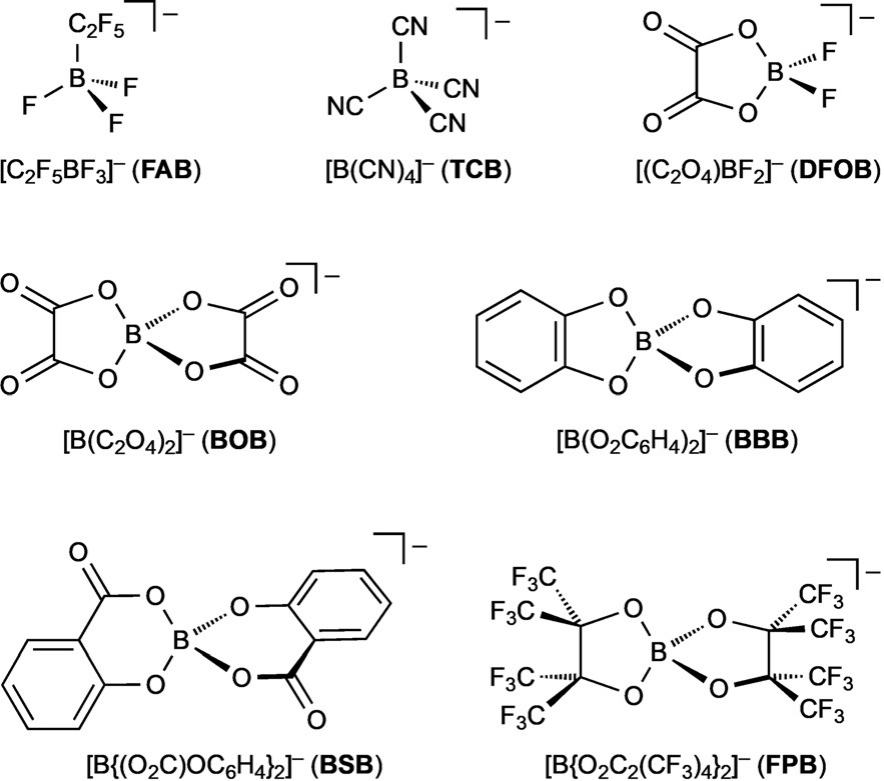
Examples of borate anions used in battery electrolytes.

Lithium tetrafluoroborate Li[BF_4_] was studied as a conducting salt for Li‐ion batteries, as an alternative to Li[PF_6_],46d but most of its properties were found to be inferior. For example, it provides a lower ion conductivity than Li[PF_6_]. Furthermore, it is hydrolytically unstable, similar to Li[PF_6_].46c, 56 In addition to the tetrafluoroborate anion, a number of other tetracoordinate borate anions have been studied for applications in battery electrolytes (Figure 7).

The replacement of the fluorine substituents of [BF_4_]^−^ by perfluoroalkyl groups led to improved properties, especially higher conductivities of the Li^+^ salts in solution. Li[C_2_F_5_BF_3_] (Li**FAB**, Figure 7) was studied in detail,46c, 56c, 56d and promising data for Li[B(CF_3_)_4_] (Figure 6) have been published.52b, 52d The reason for the higher conductivity of Li[C_2_F_5_BF_3_] and Li[B(CF_3_)_4_] compared to Li[BF_4_] is the weaker interaction between the Li^+^ cation and the borate anion. Lithium tetracyanoborate (Li**TCB**, Figure 7), an analogue of Li[BF_4_] in which all four F atoms are replaced by CN groups, was first prepared in early 2000.4b, 46d, 57 It is thermally and electrochemically very robust56b but poorly soluble in conventional solvents, thereby resulting in a low ion conductivity.4b

Lithium spiroborates have been developed as further alternatives for Li[BF_4_] and Li[PF_6_].46d, 56b Among these salts, lithium bis(oxalato)borate (Li**BOB**, Figure 7) is the most prominent fluorine‐free example. The disadvantages of Li**BOB** are its limited anodic stability and its rather low solubility in carbonate solvents. Nevertheless, Li**BOB** has significant potential for application in Li‐battery technology.4b, 56b Lithium difluoro(oxalato)borate (Li**DFOB**, Figure 7) offers a combination of the advantages of both parent salts, Li[BF_4_] and Li**BOB**, that is, thermal stability and high ionic conductivity in conventional carbonate solvents as a result of its good solubility.56b Several related lithium spiroborates have been synthesized and tested in Li‐ion batteries, for example, lithium bis(salicylato)borate (Li**BSB**) and lithium bis(1,2‐benzenediolato)borate (Li**BBB**; Figure 7).56b

Most of the aforementioned spiroborate anions, related borate anions, and some neutral boranes such as tris(pentafluorophenyl)borane (B(C_6_F_5_)_3_), have been employed as additives to electrolytes for Li‐ion batteries.56b, 58 In addition, ionomers with borate moieties have been studied as components for gel and polymer electrolytes, for example, lithium poly[oligo(ethyleneglycol)oxalato]borate.56b

Beyond Li‐ion batteries, batteries based on other metals such as sodium, magnesium, and aluminum are of growing interest.^47^, ^59^ A range of borate anions have been employed as counterions for these types of batteries. Since the demands of the battery are determined by the metal and the type of battery (e.g. metal‐oxygen, metal‐sulfur, or metal‐air battery), the borate anion has to be chosen carefully. Thus, borate anions successfully employed in Li‐ion batteries are not necessarily components of choice for other batteries. Very recently, the Mg salt of the perfluorinated bis(pinacolato)borate anion ([B{O_2_C_2_(CF_3_)_4_}_2_]^−^, **FPB**; Figure 7) was found to be a promising, chloride‐free component of electrolytes for Mg batteries.^60^

### 4.2 Low‐Viscosity Room‐Temperature Cyanoborate Ionic Liquids: Electrolyte Components for Electrochemical Devices

**Figure 8:**
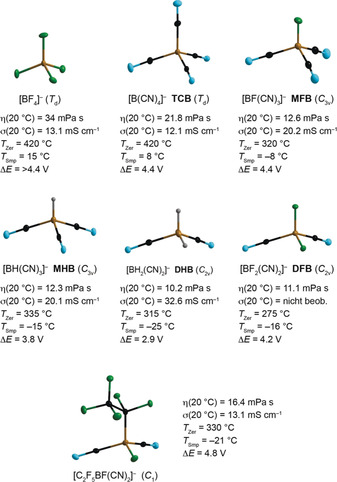
Structures of selected (cyano)borate anions and selected properties of the respective EMIm‐ILs: dynamic viscosity *η*(20 °C), specific conductivity *σ*(20 °C), melting point (*T*_m_; DSC onset), decomposition temperature (*T*_dec_; DSC onset), and electrochemical window (Δ*E*=*E*_ox_−*E*_red_).

Ionic liquids (ILs) based on the tetrafluoroborate anion [BF_4_]^−^ have been employed as electrolytes and as components of electrolytes in a wealth of different electrochemical applications,^61^ for example of Li‐ion46d, 46e, 62 and Na‐ion batteries.46e Reasons for the widespread use of tetrafluoroborate‐ILs are 1) the availability of salts of the [BF_4_]^−^ ion and 2) their beneficial properties. Many of them are room‐temperature ionic liquids (RTILs) that possess low viscosities and melting points and provide large electrochemical windows, for example, [EMIm][BF_4_] (EMIm=1‐ethyl‐3‐methylimidazolium; Figure 8).

Cyanoborate‐based ILs often have superior properties compared to the respective tetrafluoroborate‐ILs, that is, hydrophobicity, lower viscosities, and melting points.57a, 63 As mentioned above, a low dynamic viscosity is a prerequisite for electrolytes because it is paralleled by a high ion mobility, as evident from a high specific conductivity. Tetracyanoborate‐ILs (**TCB**‐ILs, Figure 7) were the first cyanoborate‐ILs that were synthesized and applied for electrochemical devices,61a, 61b, 63, 64 for example, DSSCs.49b, 49c, 49d, 49e, 65 Later, applications in batteries and supercapacitors were also studied.48e, 48f, 57a, 63 A number of further cyanoborate anions have been intensively studied in recent years and these developments have been reviewed very recently.57a, 63 Important examples are salts of fluorocyanoborate anions [BF_n_(CN)_4−*n*_]^−^ (*n*=1 (**MFB**), 2 (**DFB**), 3),^63^, ^66^ cyanohydridoborate anions [BH_*n*_(CN)_4−*n*_]^−^ (*n*=1 (**MHB**), 2 (**DHB**)),^67^ and monoperfluoroalkylcyano(fluoro)borate anions [R^F^BF_*n*_(CN)_3−*n*_]^−^ (*n*=1, 2).^68^ RTILs based on these anions have been successfully tested in different types of electrochemical devices.^63^ The dynamic viscosity of an IL depends on the mass and symmetry of the anion.^63^, ^69^ Thus, the EMIm‐IL of the highly symmetrical [B(CN)_4_]^−^ anion possesses a higher dynamic viscosity than the EMIm‐ILs of many mixed‐substituted cyanoborate anions of lower symmetry, for example, [BF(CN)_3_]^−^, [BF_2_(CN)_2_]^−^, [BH(CN)_3_]^−^, [BH_2_(CN)_2_]^−^, and [C_2_F_5_BF(CN)_2_]^−^ (Figure 8). The low viscosities of these EMIm‐ILs are accompanied by high specific conductivities and [EMIm]**DHB** has the highest specific conductivity of all non‐protic ILs known to date.^63^, ^67^

### 4.3 Anionic Boron Clusters: Building Blocks for Battery Applications

**Figure 9:**
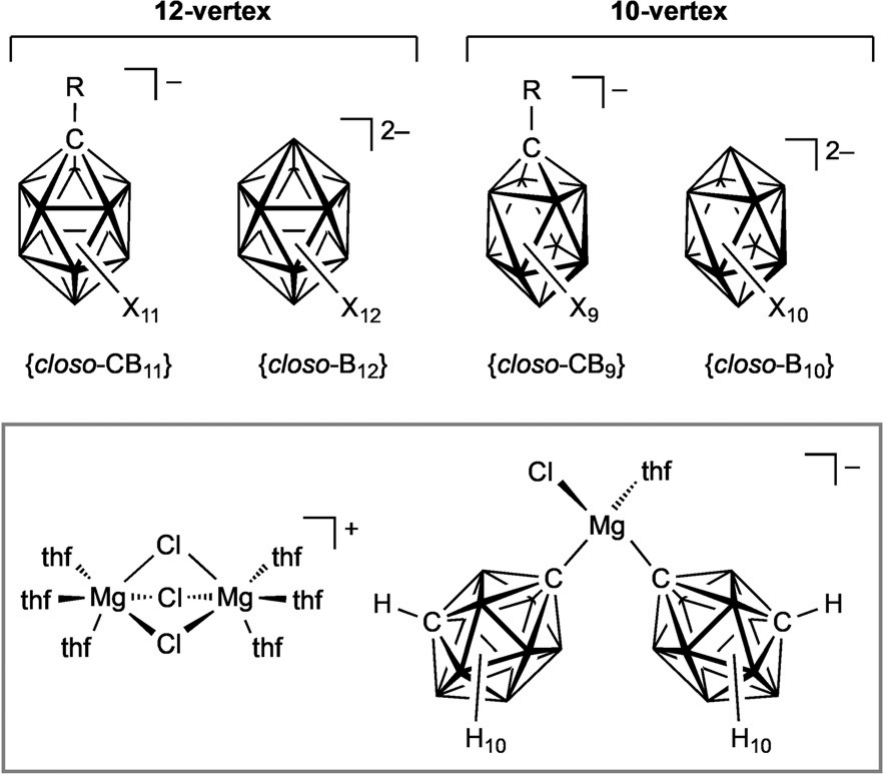
The most relevant parent boron clusters with respect to metal‐ion batteries (top) and a complex Mg^2+^ salt that contains 1,7‐carboranyl ligands (bottom, thf=tetrahydrofuran).

Negatively charged boron clusters have been tested as counteranions in lithium‐, sodium‐, and magnesium‐ion batteries.^70^ The most robust mono‐ and dianionic twelve‐vertex clusters {*closo*‐1‐CB_11_} and {*closo*‐B_12_} and to a lesser extent the related ten‐vertex derivatives {*closo*‐1‐CB_9_} and {*closo*‐B_10_} have been a focus of this research (Figure 9).47b, 70a, 71 In addition to the parent anionic boron clusters with hydrogen substituents, per‐ and polyhalogenated clusters, which are among the most weakly coordinating anions,^50^ are of interest for battery applications. In general, the introduction of halogen substituents results in increased chemical and electrochemical stability.^72^

The first studies of Li salts of polyhalogenated *closo*‐borate ions, Li_2_[*closo*‐B_10_Cl_10_] and Li_2_[*closo*‐B_12_Cl_12_], as components of electrolytes date back to the 1970s.46b, 71 Later, lithium salts of other anionic clusters were prepared and some of their properties, for example, specific conductivity, have been studied. Especially noteworthy are the per‐ and polyfluorinated derivatives46d Li_2_[*closo*‐B_12_F_12_] (Figure 9),^54^, ^73^ [(CH_3_)_3_N‐*closo*‐B_12_F_11_]^−^,^74^ and [1‐CH_3_‐*closo*‐1‐CB_11_F_11_]^−^ (Figure 9),74b which provide high specific conductivities.

The parent clusters [*closo*‐1‐CB_*n*_H_*n*+1_]^−^ (*n*=11, 9) and [*closo*‐B_*n*_H_*n*_]^2−^ (*n*=12, 10) have attracted considerable interest as halogen‐free counterions in battery applications in recent years, especially for Mg‐ion batteries.59a, 59c, 59d, 70, 71, 75 In addition to magnesium salts of all‐boron and monocarbaboron *closo*‐clusters, a Grignard‐type electrolyte based on deprotonated 1,7‐dicarba‐*closo*‐dodecaborane (*m*‐carborane) gave promising results. The salt [Mg_2_C_l3_(thf)_6_]^+^[Mg(*closo*‐1,7‐C_2_B_10_H_11_)_2_Cl(thf)]^−^ was crystallized from a solution of this electrolyte (Figure 9) and thus provided evidence for the coordination of the carboranyl ligand to the metal center through a Mg−C_cluster_ bond.^76^

Ionic liquids based on anionic boron clusters70a, 77 are promising components of electrolytes for Li‐ and Mg‐ion batteries. These ILs are replacements for volatile ethereal solvents such as tetrahydrofuran.^78^

In addition to applications in liquid electrolytes, anionic boron clusters have been discussed as building blocks for solid‐state metal‐ion batteries,70b, 70c, 70d, 70e, 70f, 71, 79 especially because of the superionic conduction observed, for example, for Na_2_[*closo*‐B_12_H_12_]^80^ and M_2_[*closo*‐1‐CB_9_H_10_][*closo*‐1‐CB_11_H_12_] (M=Li, Na).^81^ The mixed salt Na_2_[*closo*‐B_12_H_12_]_0.5_[*closo*‐B_10_H_10_]_0.5_ was used for the construction of a stable 3 V all‐solid‐state sodium‐ion battery,^82^ which exemplifies the potential of these solid electrolytes. The study of metal‐ion conductivity of salts of anionic perhalogenated *closo*‐boron clusters showed the potential of such salts for battery applications.79b

Anionic *closo*‐boron clusters with pseudohalogen substituents, for example, [*closo*‐B_12_X_12_]^2−^ and [*closo*‐1‐CB_11_X_12_]^−^ (X=CN, SCN),^83^ have been suggested as alternatives for polyhalogenated *closo*‐clusters for battery and related applications.^83^, ^84^ These clusters have been calculated to provide extraordinarily high stabilities against oxidation. This prediction was recently supported by the results of an experimental gas‐phase study on the [*closo*‐B_12_(CN)_12_]^2−^ ion.^85^ A few related mixed‐substituted {*closo*‐1‐CB_11_} anions bearing halide and cyano substituents have also been reported.^86^

### 4.4 Summary and Perspectives

Tetracoordinate borate anions and anionic boron clusters are highly important building blocks for diverse fields of materials chemistry, especially for electrochemical and optoelectronic devices, such as batteries, supercapacitors, and solar cells. Their versatility allows for facile tuning of the properties, and thus borate anions will be of increasing importance for the design of advanced conducting salts and ionomers. This will stimulate research into both metal and organic salts of boron‐based anions.

## 5 Boron Molecules in OLEDs

Organic light‐emitting diodes (OLEDs) are devices that convert electrical energy into light. Since the demonstration of low‐driving‐voltage OLEDs based on tris(8‐hydroxyquinolinato)aluminum (Alq_3_) by Tang and VanSlyke in 1987,^87^ OLED technologies have undergone tremendous progress and have found applications in a variety of consumer products. The use of boron‐based molecules for applications in OLEDs have been an active research field for more than two decades. The seminal work of Tang and VanSlyke certainly inspired much of the early research into boron compounds as emitters in OLEDs, driven by the need for stable and brighter emitters, especially blue emitters.

### 5.1 Boron‐Based Molecules in Fluorescent and Phosphorescent OLEDs

**Figure 10:**
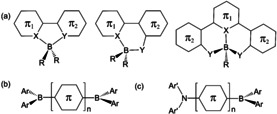
Representative structures of tri‐ and tetracoordinate boron compounds used as either fluorescent emitters or charge‐transport/blocking materials in OLEDs.

Many examples of highly efficient and stable tetracoordinate boron fluorescent emitters with emission colors ranging from blue to red have been achieved and successfully used in OLEDs. Representative structures of tetracoordinate boron compounds as fluorescent emitters for OLEDs are shown in Figure 10 a. The X and Y atoms are usually heteroatoms such as oxygen or nitrogen atoms. In some cases, the X atom can be a carbon atom of an aryl ring (e.g. phenyl). The R groups are typically aryl substituents or fluoride. The π_1_ and π_2_ rings are typically five‐ or six‐membered aryl or heteroaryl groups. Tetracoordinate boron compounds for applications in OLEDs have been covered in several reviews,^88^, ^89^, ^90^ and thus will not be described further here.

The use of three‐coordinate boron compounds, namely triarylboranes, in OLEDs was pioneered by Shirota and co‐workers.^91^ They first demonstrated the use of a series of dithienyl‐ or trithienyl‐linked bis(triarylborane) molecules, with the general structure shown in Figure 10 b, as highly efficient electron‐transport materials in OLEDs.91a This stems from the empty p_π_ orbital of the boron center, which undertakes p_π_‐π conjugation with the neighboring aryl units, hence promoting electron transport. Many other triarylboranes or boron‐embedded π‐conjugated systems have since been demonstrated to be effective electron‐transporting materials in OLEDs.^92^ Subsequent studies by Shirota and co‐workers revealed that the π‐conjugated thiophene‐bis(triarylborane) compounds are also bright blue emitters and can be used as blue emitters in OLEDs.91b The Shirota group was also the first to demonstrate the use of triarylborane compounds as hole‐blocking materials in OLEDs.91c When combining an electron‐accepting triarylboron unit with a conjugated electron donor unit such as an amino group, the resulting “bipolar” molecules shown in Figure 10 c have an intense intramolecular charge‐transfer transition from the donor to the boron center that leads to bright luminescence. The Shirota group was the first to recognize the potential of such bipolar donor–acceptor boron compounds as emitters in OLEDs. They successfully demonstrated the use of several bipolar boron compounds in efficient OLEDs with tunable emission colors from blue‐green to yellow, based on the extent of conjugation of the π‐skeleton.^93^ This early work inspired intensive subsequent research efforts into donor–acceptor‐based boron compounds for OLEDs.^94^

Another important application of triarylboranes is their use as functional groups in phosphorescent transition‐metal emitters for OLEDs. The triarylborane unit has been shown by several research teams to be highly effective in enhancing the phosphorescence quantum yields of metal complexes, thereby leading to high‐performance OLEDs. Detailed accounts are provided in several reviews/book chapters.^95^

### 5.2 Boron‐Based TADF Emitters

**Figure 11:**
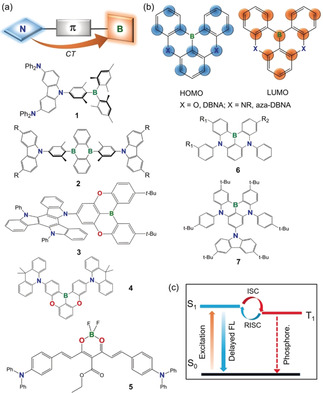
Representative examples of donor–acceptor boron‐based TADF emitters (a) and multiresonance boron‐based TADF emitters (b), as well as a diagram illustrating the difference between TADF and phosphorescence (c).

The most significant recent development in boron‐based molecules for OLEDs is their use as thermally activated delayed‐fluorescence (TADF) emitters, which is described here in detail. The seminal discovery of TADF emitters and their use in OLEDs by Adachi and co‐workers^96^ ignited intense international interest and efforts in TADF research.^97^ Like phosphorescent emitters, TADF emitters have the advantages of capturing both singlet and triplet excitons as photons in OLEDs, thus greatly increasing the internal quantum efficiency of OLEDs. This is especially important for achieving high‐efficiency blue OLEDs.^98^ Blue emitters, especially triplet blue emitters, are well‐known to suffer from low stability in the excited state and to have a relatively short operating lifetime in OLEDs because of the high excitation energy applied to these molecules.^99^ The development of blue phosphorescent emitters for OLEDs that possess high efficiency and high stability has been a longstanding challenge in OLED research and development. As illustrated in Figure 11, for phosphorescent emitters, both singlet and triplet excitons are harvested through intersystem crossing (ISC) through the phosphorescence decay channels. Therefore, to achieve blue emission (λ_phos_≈450 nm), it is necessary to excite the molecule to the singlet excited state (S1 usually) in the near‐UV region (λ_ex_<400 nm). For TADF, because both the singlet and triplet exciton excitons are harnessed through the fluorescence decay channels (λ_FL_≈450 nm) by a reverse intersystem crossing (RISC) process promoted by thermal energy, only the excitation of the molecule to the first singlet excited state is necessary. As a result, the excitation energy, in principle, could be considerably lower than that for phosphorescent emitters, which may be beneficial for achieving efficient and stable blue emitters. In addition to the possibility of addressing the stability issue of blue emitters, TADF emitters do not require the presence of precious metals in the molecules, which could greatly reduce the manufacturing cost of blue OLEDs.^98^

To achieve efficient TADF emission, the RISC process must be efficient at ambient temperature, which requires the singlet–triplet energy separation (Δ*E*_ST_) to be less than 0.2 eV. One commonly used strategy is to employ donor–acceptor systems in which the donor and the acceptor units are spatially separated with an approximate orthogonal arrangement to minimize exchange interactions and spatial overlap of the HOMO and the LUMO so that the Δ*E*_ST_ can be minimized.^96^, ^97^, ^98^, ^99^, ^100^ As triarylborane units are well‐known to be very effective and stable electron acceptors with excellent structural tunability, it is not surprising that many highly effective TADF emitters use a triarylboron moiety as the electron‐acceptor unit, and many such examples have been demonstrated recently.^101^, ^102^, ^103^, ^104^, ^105^ Representative examples of this type of boron‐containing TADF emitter (type (a)) are shown in Figure 11 a.

Boron‐based TADF emitters in this category are in fact similar to the donor–acceptor fluorescent emitters (Figure 10 c) reported by Shirota and others, but with a greater steric constraint on the donor and acceptor geometry to minimize the Δ*E*_ST_. Boron‐based TADF emitters in this system typically contain one π‐linker (most commonly used are phenyl or mesityl) between a donor unit and the boron acceptor unit. The two aryl substituents on boron are typically bulky groups such as mesityl or 2,4,6‐triisopropylphenyl groups. The donor units are usually bulky and highly constrained nitrogen donors such as carbazole or acridine (see examples **1**–**4** in Figure 11 a).^102^, ^104^, ^105^, ^106^ Highly efficient blue‐green/green OLEDs with external quantum efficiencies (EQEs) greater than 20 % have been achieved with this class of TADF emitters. It is especially noteworthy that OLEDs employing molecules **2** (R=H, **2 a**; *t*‐Bu, **2 b**) as emitters achieved record‐breaking EQEs for TADF emitters, with EQE_max_ values of 37.8 and 32.4 %, respectively.^104^ This was attributed to the flat geometry of molecules **2**, which causes alignment of their molecular axes with the OLED substrate surface upon vacuum deposition, thereby yielding thin films of the emitters with horizontally oriented dipoles. This results in an enhanced light output coupling factor,^107^ which greatly increases the EQE_max_ of the device while simultaneously decreasing its efficiency roll‐off. Recently, several examples of boron‐based TADF emitters that rely on a DBNA unit (5,9‐dioxa‐13*b*‐boranaphtho[3,2,1‐*de*]anthracene, Figure 11 b, top, with X=O) as the acceptor (e.g. **3** and **4**) have also been demonstrated to produce highly efficient blue or sky‐blue OLEDs.^105^, ^106^ In particular, compound **3**, which contains three carbazole donor groups, has been demonstrated to produce exceptionally bright and efficient blue OLEDs with an EQE_max_ of 38.15 %±0.42 % and a low efficiency roll‐off.^106^ In addition to the intrinsically high emission quantum efficiency of **3**, its flat and highly rigid structure, which leads to its horizontal orientation on the substrate, is believed to greatly enhance the light output coupling and the overall EQE of the OLEDs.^106^

The second type of boron‐based TADF emitters (Figure 11 b) was discovered by Hatakeyama and co‐workers.^108^, ^109^, ^110^ In contrast to type (a) molecules and the commonly used donor–acceptor TADF emitters, in type (b) boron TADF emitters, the donor and acceptor units are not physically located at different parts of the molecule. Instead, the donor and the acceptor units in type (b) molecules are on the same domain of the molecule, which has a highly rigid, fully conjugated/aromatic structure. However, as illustrated in Figure 11 b, the HOMO and the LUMO in this type of molecules involve contributions from two different sets of atoms, albeit on the same domain, which leads to a spatial separation of the HOMO and LUMO and a small Δ*E*_ST_. As the atoms in the HOMO and the LUMO of this type of molecule exhibit opposite resonance features, Hatakeyama and co‐workers defined this type of TADF emitter as multiple resonance effect (or multiresonance effect) emitters.^108^ Multiresonance TADF emitters have several key advantages over the classic donor–acceptor emitters. Firstly, because the emission in multiresonance emitters originates from a highly rigid π‐conjugated/aromatic unit, it has a much higher oscillator strength, thus they are often much more efficient than type (a) emitters, which are well‐known to have a very small oscillator strength and a broad charge‐transfer emission maximum, owing to the orthogonal geometry of the donor and the acceptor units. Secondly, type (b) emitters have very small Stokes shifts, a much narrower emission band with the full‐width at half‐maxima (FWHM) typically much less than 50 nm, which is highly beneficial for achieving OLEDs with a higher color purity, compared to the type (a) emitters.^109^ A recent computational study by Olivier and co‐workers using highly correlated quantum‐chemical calculations indicated that the unique TADF features displayed by type (b) boron emitters can be ascribed to the short‐range reorganization of the electron density that takes place upon electronic excitation of the multiresonant structures.^111^

The multiresonance effect was first demonstrated by Hatakeyama and co‐workers for the DBNA molecule shown in Figure 11 b.^108^ The subsequent study of related aza‐DBNA molecules by Hatakeyama and co‐workers demonstrated that substitution by a donor group at a position *para* to boron can greatly enhance the multiresonance effect of the molecule and its performance in OLEDs.^109^ For example, replacing R_2_ in **6** by a diphenylamino group produced a pure blue TADF emitter that has an EQE_max_ of 20.2 %, much higher than that based on the analogue without the amino substituent (EQE_max_=13.5 %). Introducing a carbazole donor at the alternative *para* position leads to molecule **7**. The blue OLEDs based on **7** exhibit an EQE_max_ of 32.1 % and a highly suppressed efficiency roll‐off.^112^

The third type of boron‐based TADF emitters (type (c)) involve tetracoordinate boron molecules with structural features similar to those depicted in Figure 10 a, except with the inclusion of an amino/N‐heterocyclic donor group in the aryl substituents (R). Several examples of type (c) TADF emitters have been reported recently, which achieve TADF properties by taking advantage of the charge‐transfer transition from the donor‐appended aryl groups to the π‐conjugated chelate backbone.^113^, ^114^, ^115^, ^116^ The performance of OLEDs based on tetracoordinate boron TADF emitters is generally not as impressive as the triarylboron‐based emitters shown in Figure 11. A notable example was an efficient NIR TADF emitter based on a boron difluoride curcuminoid complex (**5**) reported recently by Adachi and co‐workers.^116^ The OLEDs based on this NIR emitter display EQE_max_ values near 10 % and tunable λ_em_ from 700 to 780 nm, which is very impressive for NIR OLEDs.

### 5.3 Summary and Perspectives

In summary, the recent discoveries of boron‐based TADF emitters demonstrate that the incorporation of a boron unit can greatly improve the performance of OLEDs. Boron‐based molecules will likely play an important role in practical and high‐performance OLEDs, especially blue OLEDs.

## 6 Conclusion

The rich chemistry of boron is evidenced by its ability to form a myriad of molecules that are proving to be highly interesting in research related to energy conversion and storage. Boron forms unique interactions with itself through single, double, or triple bonds, or with other elements such as in FLPs, all of which allow effective activation of small molecules. The combination of boron with hydrogen leads to a wide range of hydrogen‐rich molecules that hold potential as hydrogen‐storage materials. As a consequence of its electron‐deficient nature, boron forms a series of highly chemically, electrochemically, and thermally stable anions and negatively charged clusters. The anionic boron clusters feature unique multicentered bonding, and are currently finding applications as building blocks for various materials such as very low viscosity room‐temperature ionic liquids (RTILs) used in electrochemical devices. Boron‐bearing molecules also enable rich donor–acceptor tunability, which is critical to obtain OLEDs with high efficiencies and light of desired wavelengths.

The impressive recent advances in the synthesis of novel boron‐containing molecules and the diverse materials derived therefrom, combined with a significantly improved understanding of the properties of these boron‐based molecules and materials, provides a powerful opportunity for the further exploration of boron as a key element in the field of energy research.

## Conflict of interest

The authors declare no conflict of interest.

## Biographical Information

*Zhenguo Huang obtained his Ph.D. from the University of Wollongong*, *Australia. He is currently an Associate Professor at the University of Technology Sydney. His research group works on boron chemistry for energy conversion and storage, including hydrogen storage materials, electrolytes, and 2D boron‐containing nanosheets for catalysis. He was awarded a Discovery Early Career Research Award and Future Fellowships from the Australian Research Council, and is a recipient of the Humboldt Research Fellowship for Experienced Researchers*.

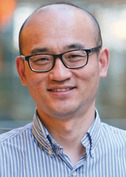

## Biographical Information

*Rian Dewhurst obtained his Ph.D. in 2006 from the Australian National University under the Supervision of Prof. Anthony F. Hill. After postdoctoral research at the University of California, Riverside with Prof. Guy Bertrand, he took up an Alexander von Humboldt Postdoctoral Fellowship at the Julius‐Maximilians‐Universität Würzburg hosted by Prof. Holger Braunschweig. He is currently a Senior Scientist in the Institute for Inorganic Chemistry and the Institute for Sustainable Chemistry & Catalysis with Boron at the Julius‐Maximilians‐Universität Würzburg*.

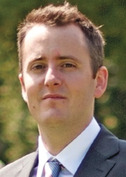

## Biographical Information

*Suning Wang obtained her Ph.D. in Chemistry from Yale University, USA. She is currently a Professor at the Department of Chemistry, Queen's University, Canada. She is an international leader in boron chemistry, especially in boron‐based optoelectronic materials and the photochemistry of boron compounds. She is a Fellow of the Royal Society of Canada and a Fellow of the Royal Society of Chemistry (UK)*.

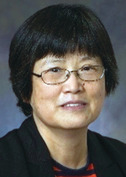

## Biographical Information

*Nikolai V. Ignat′ev (Ignatiev, Ignatyev) was born in the Pskov region, Russia in 1950. He earned his Ph.D. in 1980 and was promoted to Senior Scientist at the Institute of Organic Chemistry, Kiev, Ukraine, in 1988. In 1994–2000 he was a visiting scientist at the Universität Duisburg with Prof. Peter Sartori. Since 2000 he has been working for Merck KGaA (Darmstadt, Germany), first as a Senior Scientist and since 2015 as a Consultant. His research interests concern the chemistry and electrochemistry of organofluorine compounds, electrochemical fluorination, catalysis, ionic liquids, and weakly coordinating anions*.

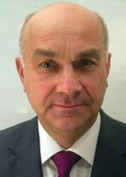

## Biographical Information

*Maik Finze studied chemistry at the University of Hannover and Stanford University. He completed his Ph.D. in 2004 at the University of Hannover in the group of Prof. H. Willner. In 2004 he started his independent research at the University of Düsseldorf in the area of boron and fluorine chemistry and related fields of materials science. In 2011 he was appointed Professor at the Julius‐Maximilians‐Universität Würzburg, where he is currently Chair of Inorganic Chemistry and Co‐Head of the Institute for Sustainable Chemistry & Catalysis with Boron (ICB)*.

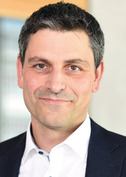

## Biographical Information

*Holger Braunschweig studied chemistry and began his independent research career at RWTH Aachen, where he obtained his PhD and Habilitation with Prof. P. Paetzold. He also performed postdoctoral research with Prof. Michael Lappert, FRS, at the University of Sussex and held a position as Reader at Imperial College, London. He is now Chair and Head of Inorganic Chemistry at the Julius‐Maximilians‐Universität Würzburg, where he is also a member of the Senate and Founding Director of the Institute for Sustainable Chemistry & Catalysis with Boron (ICB)*.